# Isolation and Anti-Melanogenic Activity of Polycarpol from *Ganoderma lucidum* Mycelia: Expanding the Functional Potential of Cultured Mycelial Biomass

**DOI:** 10.3390/jof12080625

**Published:** 2026-08-19

**Authors:** Che-Hwon Park, Sung-Chul Lee, Rae-Won Kang, Young-Jin Park

**Affiliations:** 1 Department of Medicinal Biosciences, College of Biomedicinal and Health Science, Konkuk University, Chungju 27478, Republic of Korea; chehwon9798@kku.ac.kr (C.-H.P.); vwm963@kku.ac.kr (S.-C.L.); fodnjs72617@kku.ac.kr (R.-W.K.); 2 Natural Product Bio-Convergence Research Center, Konkuk University, Chungju 27478, Republic of Korea

**Keywords:** *Ganoderma lucidum*, mycelia, polycarpol, triterpenoid, melanogenesis, tyrosinase, melanin, MITF, CREB, MAPK

## Abstract

*Ganoderma lucidum* is a medicinal mushroom widely recognized as a source of bioactive metabolites, particularly triterpenoids. Although most studies have focused on fruiting bodies and spores, the potential of cultured mycelial biomass as a source of functional secondary metabolites remains less explored. In this study, we isolated and characterized polycarpol from the mycelia of *G. lucidum* and evaluated its anti-melanogenic activity in B16F10 murine melanoma cells. Dried mycelia obtained after liquid culture were extracted with 70% ethanol, partitioned with ethyl acetate, and purified by repeated chromatographic separation. The isolated compound was identified as polycarpol by LC–ESI–MS, HR-MS/MS, and NMR spectroscopy, together with comparison with previously reported spectroscopic data. The anti-melanogenic activity of polycarpol was evaluated in α-MSH-stimulated B16F10 murine melanoma cells, a commonly used cellular model of melanogenesis. Polycarpol reduced α-melanocyte-stimulating hormone-induced cellular tyrosinase activity and melanin production at concentrations that did not markedly affect cell viability. Notably, 2 μg/mL (4.54 μM) polycarpol showed a melanin-inhibitory effect comparable to that of arbutin. Western blot analysis showed that polycarpol decreased the expression of microphthalmia-associated transcription factor, tyrosinase, and tyrosinase-related protein-1. In addition, polycarpol suppressed CREB phosphorylation and modulated the MAPK signaling pathways. These findings suggest that polycarpol inhibits melanogenesis through regulation of MITF-associated melanogenic signaling. Overall, this study demonstrates that cultured *G. lucidum* mycelia can produce anti-melanogenic triterpenoids and highlights cultured mycelial biomass as a controllable fungal material for exploring anti-melanogenic triterpenoids, although further optimization is required to improve production efficiency.

## 1. Introduction

*Ganoderma lucidum*, commonly known as Lingzhi or Reishi, is one of the most extensively studied medicinal mushrooms and has long been used in East Asian traditional medicine. Its pharmacological relevance has generally been attributed to diverse secondary metabolites and macromolecules, particularly triterpenoids and polysaccharides. Triterpenoids from *Ganoderma* species are predominantly lanostane-type compounds, and numerous studies have reported their antioxidant, anti-inflammatory, antitumor, hepatoprotective, and immunomodulatory activities. Comprehensive reviews have emphasized that *Ganoderma* triterpenoids represent one of the major chemical classes responsible for the biological properties of this medicinal fungus [1,2]. In addition to *Ganoderma* species, other basidiomycetes have also been investigated as sources of bioactive small-molecule metabolites. For instance, triterpenoids have been isolated from *Laetiporus sulphureus,* indicating that medicinal mushrooms can produce diverse bioactive compounds beyond polysaccharides and proteins [3].

Most previous investigations on *G. lucidum* bioactive compounds have focused on fruiting bodies and spores, which are the most commonly used materials in traditional and commercial applications [4]. Fruiting bodies, in particular, have been regarded as a major source of structurally diverse triterpenoids, while spores have also been reported to contain bioactive triterpenoid constituents [5,6]. However, the fruiting-body-based production system requires relatively long cultivation periods and controlled environmental conditions. In contrast, fungal mycelia can be produced under liquid-culture conditions with shorter production cycles, higher process controllability, and potentially more consistent biomass quality. Therefore, determining whether *G. lucidum* mycelia can biosynthesize useful bioactive compounds is important for expanding the utilization of this medicinal mushroom beyond conventional fruiting-body-based materials.

Although the mycelial stage has historically received less attention than fruiting bodies, recent phytochemical studies suggest that *G. lucidum* mycelia can also serve as a source of diverse secondary metabolites [7,8]. For example, lanostane-type triterpenoids have been isolated from the mycelial mat of *G. lucidum*, and their biological activities have been evaluated, supporting the idea that cultured mycelial biomass is not merely a vegetative growth form but a metabolically active material capable of producing functional compounds [7,8]. Nevertheless, compared with fruiting bodies, the chemical and biological potential of *G. lucidum* mycelia remains insufficiently characterized. Further studies are needed to identify specific mycelium-derived compounds and to evaluate their biological activities in relevant cell-based systems.

Melanogenesis is the biological process responsible for melanin production in melanocytes. While melanin plays an essential role in protecting the skin from ultraviolet radiation, excessive melanin accumulation can lead to hyperpigmentation and various cosmetic and dermatological disorders. The melanogenic process is mainly regulated by tyrosinase, tyrosinase-related protein 1, and tyrosinase-related protein 2, whose expression is controlled by microphthalmia-associated transcription factor (MITF) [9]. In α-melanocyte-stimulating hormone (α-MSH)-stimulated melanocytes and B16F10 murine melanoma cells, the cAMP/CREB/MITF axis is a major signaling route that promotes the expression of melanogenic enzymes and increases melanin synthesis. B16F10 murine melanoma cells are widely used as an experimental model for evaluating α-MSH-induced melanogenesis because they actively produce melanin and respond to melanogenic stimulation through MITF-associated signaling pathways. Therefore, this cell model is widely used to evaluate anti-melanogenic activity by measuring tyrosinase activity, melanin content, and melanogenesis-related protein expression. Several natural compounds have been reported to suppress melanogenesis by reducing tyrosinase activity, melanin content, and MITF-associated signaling pathways [10,11].

In addition to the CREB/MITF pathway, mitogen-activated protein kinase signaling pathways are involved in the regulation of melanogenesis. ERK, JNK, and p38 MAPK signaling can influence MITF stability or transcriptional activity depending on the cellular context and stimulus. Recent studies have shown that modulation of MAPK-related signaling can alter melanogenic responses, and interruption of the p38 MAPK–MSK1–CREB–MITF pathway has been suggested as a potential strategy for controlling hyperpigmentation [12,13]. These findings indicate that compounds targeting MITF-related melanogenic signaling may have potential as anti-melanogenic agents.

In the present study, we investigated whether liquid-cultured *G. lucidum* mycelia can serve as a source of anti-melanogenic secondary metabolites. A major active compound was isolated from *G. lucidum* mycelia through activity-guided fractionation combined with HPLC monitoring and was characterized using LC–ESI–MS, HR-MS/MS, and NMR spectroscopy. Based on the spectroscopic analyses, the isolated compound was finally identified as polycarpol. Its anti-melanogenic effects were evaluated in α-MSH-stimulated B16F10 cells by measuring cell viability, tyrosinase activity, melanin content, melanogenesis-related protein expression, and CREB/MAPK signaling.

## 2. Materials and Methods

### 2.1. Fungal Material and Mycelial Culture

*Ganoderma lucidum* ASI 7071 was obtained from the Mushroom Research Division, National Institute of Horticultural and Herbal Science, Rural Development Administration, Eumseong, Republic of Korea. The strain was cultured on potato dextrose agar (PDA; BD Difco, Sparks, MD, USA) at 30 °C for 15 days. For mycelial biomass production, actively growing mycelia were inoculated into 500 mL Erlenmeyer flasks containing 250 mL of potato dextrose broth (PDB; BD Difco, Sparks, MD, USA) and incubated at 30 °C for 30 days. The harvested mycelia were used for subsequent extraction and purification.

### 2.2. Extraction and Solvent Partitioning of G. lucidum Mycelia

Dried mycelia of *G. lucidum* obtained after 30 days of liquid culture were mechanically disrupted by grinding with liquid nitrogen. The pulverized mycelial powder was extracted with 70% ethanol (SK chemicals, Seongnam, Republic of Korea) under reflux at 70 °C for 2 h, and the extraction process was repeated twice. The combined extracts were filtered under reduced pressure to remove insoluble residues and then concentrated in vacuo to obtain a crude ethanol extract.

The crude extract was re-dissolved in distilled water and partitioned with an equal volume of ethyl acetate (SK chemicals, Seongnam, Republic of Korea). The ethyl acetate-soluble fraction was collected and concentrated under reduced pressure to yield the EtOAc-soluble fraction for subsequent chromatographic purification.

### 2.3. Fractionation and Isolation of Polycarpol

**Figure 1:**
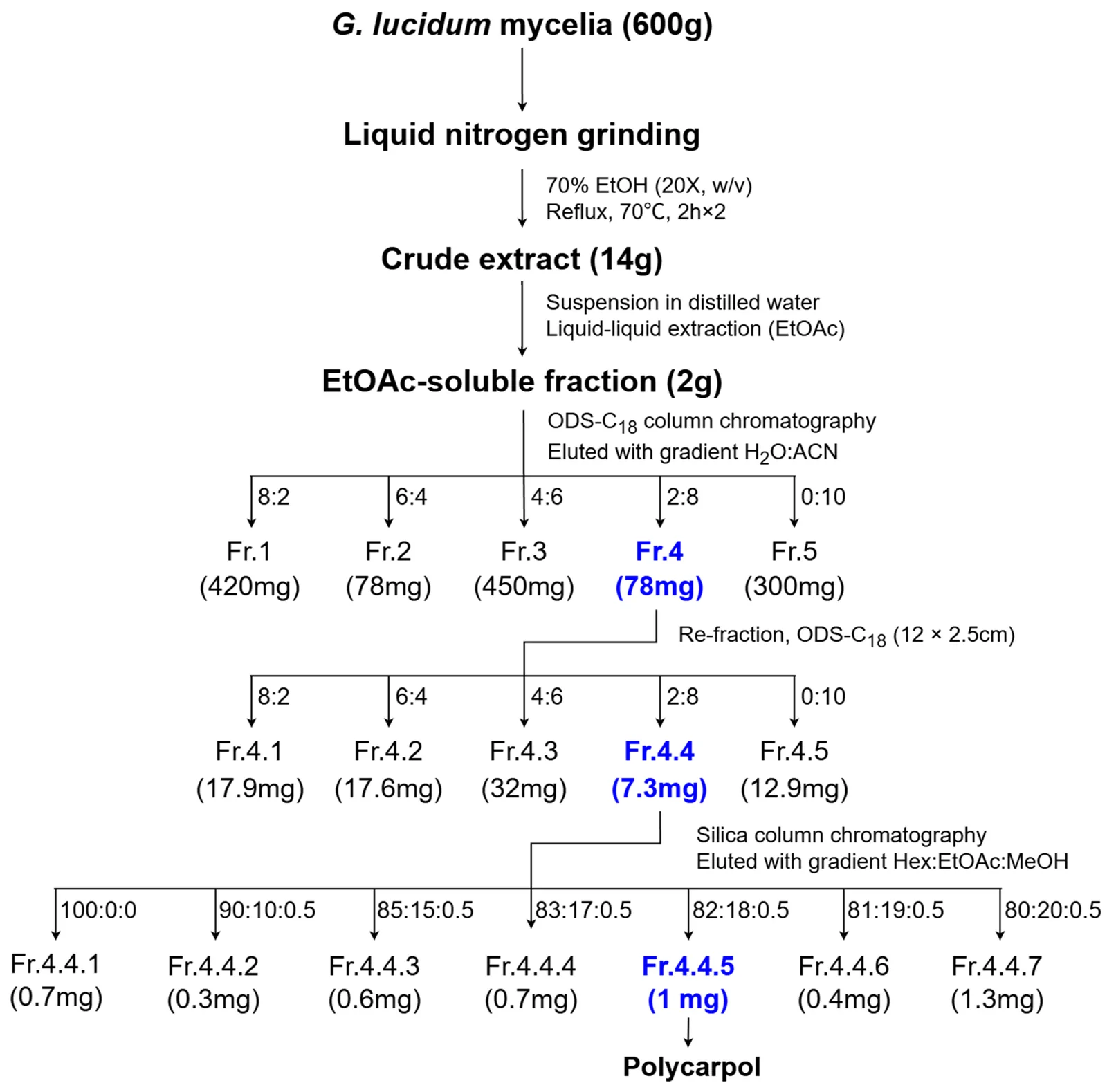
Isolation workflow of the target compound from cultured *G. lucidum* mycelia. Dried mycelia were extracted with 70% ethanol, and the crude extract was partitioned with ethyl acetate. The EtOAc-soluble fraction was separated by reversed-phase C18 column chromatography to obtain Fr.1–Fr.5. Fr.4 was further fractionated into Fr.4.1–Fr.4.5, and Fr.4.4 was purified by silica gel column chromatography to yield Fr.4.4.1–Fr.4.4.7. The target compound was obtained from Fr.4.4.5 (polycarpol).

The EtOAc-soluble fraction was subjected to reversed-phase C18 column chromatography (Merck KGaA, Darmstadt, Germany). Elution was performed using a stepwise gradient of water/acetonitrile mixtures of 8:2, 6:4, 4:6, 2:8, and 0:10 (*v*/*v*), yielding five fractions designated as Fr.1–Fr.5. The yields of the fractions obtained during the purification process are shown in Figure 1. Among these fractions, Fr.4, eluted with water/acetonitrile at 2:8 (*v*/*v*), was selected for further purification based on chromatographic analysis.

Fr.4 was further separated by reversed-phase C18 column chromatography using the same water/acetonitrile gradient system of 8:2, 6:4, 4:6, 2:8 and 0:10 (*v*/*v*), yielding subfractions Fr.4.1–Fr.4.5. Among these, Fr.4.4 was selected for additional purification. Fr.4.4 was then purified by normal-phase silica gel column chromatography (Merck KGaA, Darmstadt, Germany) using a gradient system of hexane/ethyl acetate/methanol: 100:0:0, 90:10:0.5, 85:15:0.5, 83:17:0.5, 82:18:0.5, 81:19:0.5, and 80:20:0.5 (*v*/*v*/*v*). This separation yielded fractions Fr.4.4.1–Fr.4.4.7, and the target compound was obtained from Fr.4.4.5. The purified compound was subsequently subjected to spectroscopic analysis for structural elucidation. At each major purification step, fractions were screened for anti-melanogenic activity using B16F10 cells, and the fraction showing the strongest activity was selected for further purification. HPLC analysis was used to monitor chromatographic profiles and the enrichment of the major active compound. Based on this activity-guided and HPLC-monitored purification strategy, Fr.4.4.5 was selected for spectroscopic characterization and subsequent biological evaluation.

### 2.4. HPLC Analysis of Extracts and Fractions

HPLC analysis was performed using an Alliance 2690 HPLC system (Waters Corporation, Milford, MA, USA) to monitor the chromatographic profiles of the G. lucidum mycelial extract, solvent fractions, and purified fraction. Chromatographic separation was carried out using a reversed-phase C18 column (250 × 4.6 mm, 5 μm particle size; Phenomenex Co., Torrance, CA, USA). The mobile phase consisted of solvent A, water containing 0.2% acetic acid, and solvent B, acetonitrile (J.T.Baker, Avantor, Radnor, PA, USA). Gradient elution was performed from 80% solvent A and 20% solvent B to 100% solvent B over 100 min. The detailed gradient program is shown in Table S1. The purity of the isolated compound was evaluated by HPLC using peak area normalization at 254 nm.

### 2.5. LC–ESI–MS Analysis

LC–ESI–MS analysis of the isolated compound was performed using an Agilent 1260 Infinity LC system coupled with an Agilent 6125B single quadrupole mass spectrometer equipped with an electrospray ionization source. Chromatographic separation was achieved on a reversed-phase C18 column with dimensions of 250 × 4.6 mm and a particle size of 5 μm. The column temperature was maintained at 40 °C.

The mobile phase consisted of solvent A, water containing 0.2% acetic acid, and solvent B, acetonitrile. The flow rate was set at 0.8 mL/min, and the injection volume was 10 μL. Mass spectrometric detection was performed in positive electrospray ionization mode over an *m*/*z* range of 100–1000. The ion source parameters were as follows: gas temperature, 350 °C; drying gas flow, 12 L/min; nebulizer pressure, 35 psig; and capillary voltage, 4 kV. Data acquisition and analysis were carried out using OpenLAB CDS ChemStation Edition C.01.10 (Agilent Technologies, Santa Clara, CA, USA).

### 2.6. High-Resolution Mass Spectrometry and MS/MS Analysis

High-resolution mass spectrometry was performed to determine the accurate molecular weight and molecular formula of the isolated compound. HR-MS and MS/MS analyses were conducted at the Korea Basic Science Institute using an ACQUITY UPLC I-Class system (Waters, Milford, MA, USA) coupled with a timsTOF fleX MALDI-2 mass spectrometer (Bruker Daltonics, Bremen, Germany) positive electrospray ionization mode. Chromatographic separation was performed on a reversed-phase C18 column using a mobile phase composed of water containing 0.2% acetic acid and acetonitrile. The mass scan range was set at *m*/*z* 100–1000, and both HR-MS and MS/MS spectra were acquired to characterize the molecular ion and major fragment ions.

### 2.7. NMR Spectroscopic Analysis

NMR spectroscopic analysis was performed using a Bruker Avance II 800 MHz nuclear magnetic resonance spectrometer (Bruker Corporation, Billerica, MA, USA) at the Korea Basic Science Institute, Ochang Center, Cheongju, Republic of Korea. The purified compound was dissolved in deuterated chloroform. All NMR experiments were conducted at room temperature.

^1^H and ^13^C NMR spectra were recorded at 800 MHz and the corresponding carbon frequency, respectively. DEPT-135 and two-dimensional HSQC, COSY, and HMBC experiments were performed using standard pulse sequences provided by the instrument manufacturer. Chemical shifts were reported in parts per million relative to the residual solvent signals of CDCl_3_ at δ_H_ 7.26 ppm and δ_C_ 77.0 ppm. Data acquisition and processing were performed using the NMR data were processed using Mnova software (version 17.0.0; Mestrelab Research, Santiago de Compostela, Spain).

### 2.8. Cell Culture and Viability Assay

B16F10 murine melanoma cells were purchased from the American Type Culture Collection (ATCC, Manassas, VA, USA). The cells were maintained in Dulbecco’s Modified Eagle’s Medium (DMEM; Thermo Fisher Scientific Inc., Waltham, MA, USA) supplemented with 10% fetal bovine serum (FBS; Thermo Fisher Scientific Korea, Seoul, Republic of Korea) and 1% penicillin (Thermo Fisher Scientific Korea, Seoul, Republic of Korea). Cells were incubated at 37 °C in a humidified atmosphere containing 5% CO_2_ and subcultured regularly before reaching complete confluence.

The cytotoxic effect of polycarpol on B16F10 cells was evaluated using the MTT assay [14]. B16F10 cells were seeded in culture plates and stabilized under standard culture conditions. The cells were treated with polycarpol at the indicated concentrations for 72 h. MTT solution (Sigma-Aldrich, St. Louis, MO, USA) was then added, and the cells were incubated for an additional 3 h. After removal of the medium, the intracellular formazan crystals were dissolved in dimethyl sulfoxide (DMSO; Sigma-Aldrich, St. Louis, MO, USA). Absorbance was recorded at 570 nm using a microplate reader (TECAN, Männedorf, Switzerland). Cell viability was expressed as a percentage of the untreated control.

### 2.9. Cellular Tyrosinase Activity and Melanin Content Assays

Cellular tyrosinase activity and melanin content were evaluated using B16F10 murine melanoma cells treated with polycarpol.

For the cellular tyrosinase activity assay, B16F10 cells were treated with polycarpol in the presence of 100 nM α-melanocyte-stimulating hormone (α-MSH; Sigma-Aldrich, St. Louis, MO, USA) for 72 h. After treatment, the cells were harvested and lysed with phosphate-buffered saline (PBS, pH 6.8) containing 1% Triton X-100 (Sigma-Aldrich, St. Louis, MO, USA). The lysates were centrifuged at 10,000× *g* for 10 min, and the protein concentration of each supernatant was determined using Bradford reagent (Bio-Rad Korea, Seoul, Republic of Korea).

For the enzymatic reaction, 40 μg of cellular protein was added to a 96-well flat-bottom plate containing 2.0 mM L-DOPA (Merck Korea, Seoul, Republic of Korea) and 0.1 M PBS (pH 6.8). The reaction mixture was incubated at 37 °C for 1 h, and absorbance was measured at 475 nm using a microplate reader (TECAN, Männedorf, Switzerland). Cellular tyrosinase activity was calculated relative to the α-MSH-treated control.

For the melanin content assay, B16F10 cells were treated with polycarpol for 72 h. The cells were washed with PBS and then incubated in 1 M sodium hydroxide at 60 °C for 1 h to dissolve cellular melanin. The absorbance was measured at 405 nm using a microplate reader (TECAN, Männedorf, Switzerland). Relative melanin content was expressed as a percentage of the α-MSH-treated control.

### 2.10. Western Blot Analysis

The effects of polycarpol on melanogenesis-related proteins and signaling molecules were evaluated by Western blot analysis. After treatment, B16F10 cell pellets were lysed in radioimmunoprecipitation assay buffer (RIPA; Thermo Fisher Scientific, Waltham, MA, USA). The lysates were centrifuged at 10,000× *g* for 10 min, and protein concentrations were determined using Bradford reagent (Bio-Rad Korea, Seoul, Republic of Korea).

Equal amounts of protein were resolved by sodium dodecyl sulfate-polyacrylamide gel electrophoresis and transferred to Immobilon polyvinylidene fluoride membranes (Merck KGaA, Darmstadt, Germany). Membranes were blocked with 3% bovine serum albumin and then incubated at 4 °C for 18 h with primary antibodies against tyrosinase, tyrosinase-related protein-1, microphthalmia-associated transcription factor, p38, phospho-p38, extracellular signal-regulated kinase, phospho-extracellular signal-regulated kinase, c-Jun N-terminal kinase, phospho-c-Jun N-terminal kinase, cAMP response element-binding protein, and phospho-cAMP response element-binding protein. All primary antibodies were diluted 1:1000 and obtained from Cell Signaling Technology (Danvers, MA, USA).

The membranes were subsequently incubated with horseradish peroxidase-conjugated secondary antibodies diluted 1:5000 (Cell Signaling Technology). Protein bands were detected using a C-DiGit Blot Scanner (LI-COR Korea, Gyeonggi-do, Republic of Korea), and band intensities were quantified relative to the corresponding loading or total protein controls.

### 2.11. Statistical Analysis

All experiments were performed in three independent biological replicates, with each condition measured in triplicate unless otherwise stated. Data are presented as the mean ± standard deviation. Data are presented as the mean ± standard deviation. Statistical analyses were performed using GraphPad Prism 5.04 (GraphPad Software, San Diego, CA, USA). Statistical differences among groups were evaluated by one-way analysis of variance followed by Tukey’s multiple comparison test. Differences were considered statistically significant at *p* < 0.05. Statistical significance is indicated as * *p* < 0.05, ** *p* < 0.01, and *** *p* < 0.001 versus the untreated control or α-MSH-treated control, as indicated in each figure legend.

## 3. Results

### 3.1. Isolation and Chromatographic Purification of a Major Compound from G. lucidum Mycelia

To investigate whether cultured *G. lucidum* mycelia can serve as a source of bioactive secondary metabolites, dried mycelial biomass obtained after 30 days of liquid culture was extracted and subjected to chromatographic purification. The mycelia were extracted with 70% ethanol, and the resulting crude extract was partitioned with ethyl acetate to obtain the EtOAc-soluble fraction. The EtOAc-soluble fraction was then separated by reversed-phase C18 column chromatography using a stepwise water/acetonitrile gradient, yielding five fractions designated Fr.1–Fr.5. Among these fractions, Fr.4 was selected for further purification because it retained anti-melanogenic activity in the screening assay. Fr.4 was subsequently separated into Fr.4.1–Fr.4.5, and Fr.4.4 was further purified by normal-phase silica gel column chromatography to obtain Fr.4.4.1–Fr.4.4.7. Finally, the target compound was obtained from Fr.4.4.5 and subjected to spectroscopic analysis for structural identification. The yield of each fraction, including the final Fr.4.4.5 fraction, is indicated in the purification workflow shown in Figure 1.

**Figure 2:**
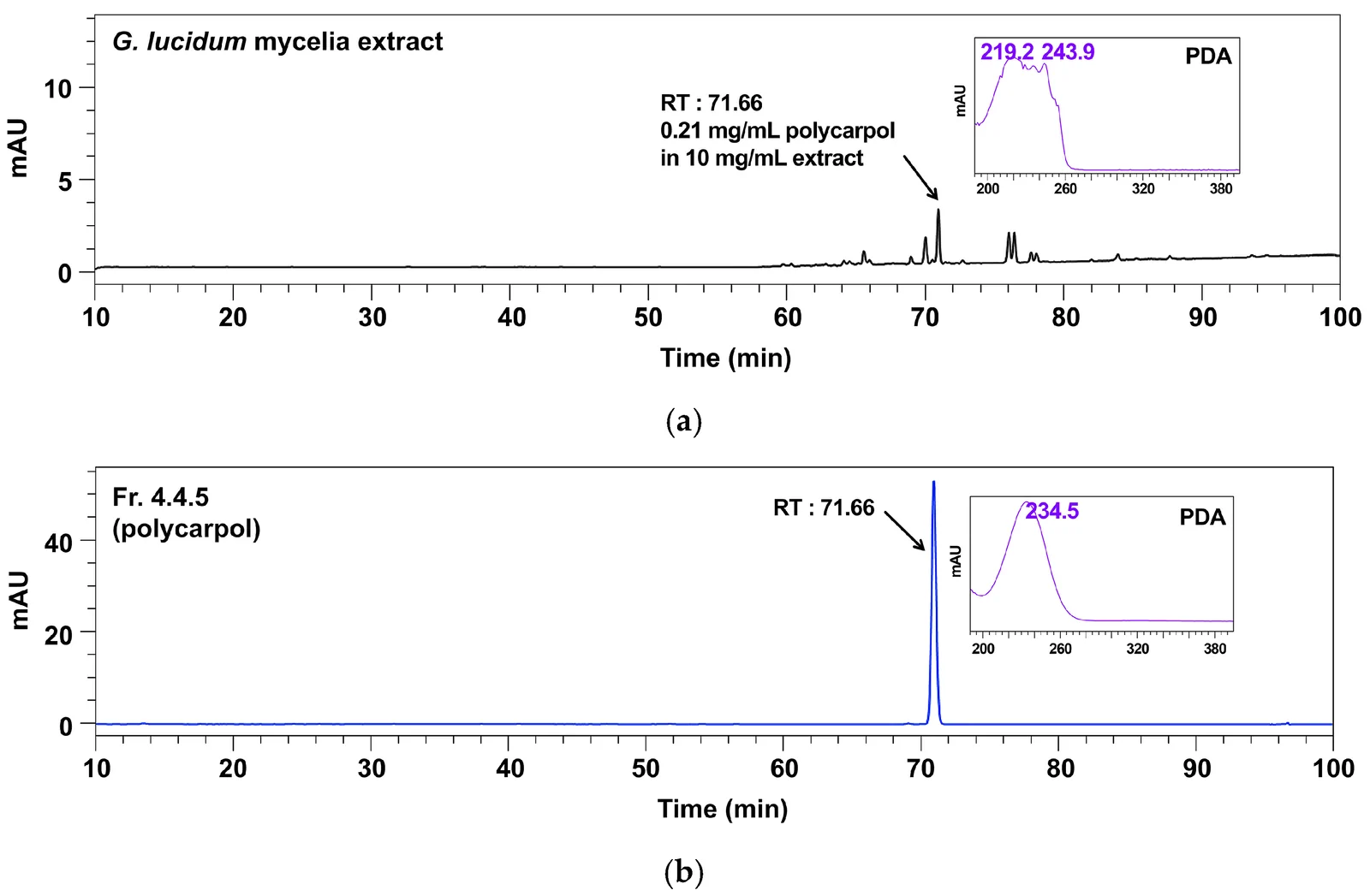
HPLC-based purification of the target compound from *G. lucidum* mycelia. Representative HPLC chromatograms monitored at 254 nm comparing the crude mycelial extract (**a**) and the final purified fraction (**b**).

HPLC analysis was used to monitor the purification process from the crude extract to the final purified fraction. Compared with the crude extract, the EtOAc-soluble fraction showed enrichment of several relatively hydrophobic peaks, indicating that solvent partitioning effectively concentrated EtOAc-soluble constituents. Subsequent C18 fractionation further simplified the chromatographic profiles, and Fr.4 displayed a more defined peak pattern than the crude extract and EtOAc-soluble fraction. Further purification of Fr.4.4 resulted in the enrichment of a dominant chromatographic peak in Fr.4.4.5, suggesting that repeated chromatographic separation successfully concentrated the target compound from the mycelial extract (Figure 2). The purity of the isolated polycarpol in Fr.4.4.5 was estimated to be 98% based on HPLC peak area normalization.

### 3.2. LC–ESI–MS and HR-MS/MS Analysis of the Isolated Compound

**Figure 3:**
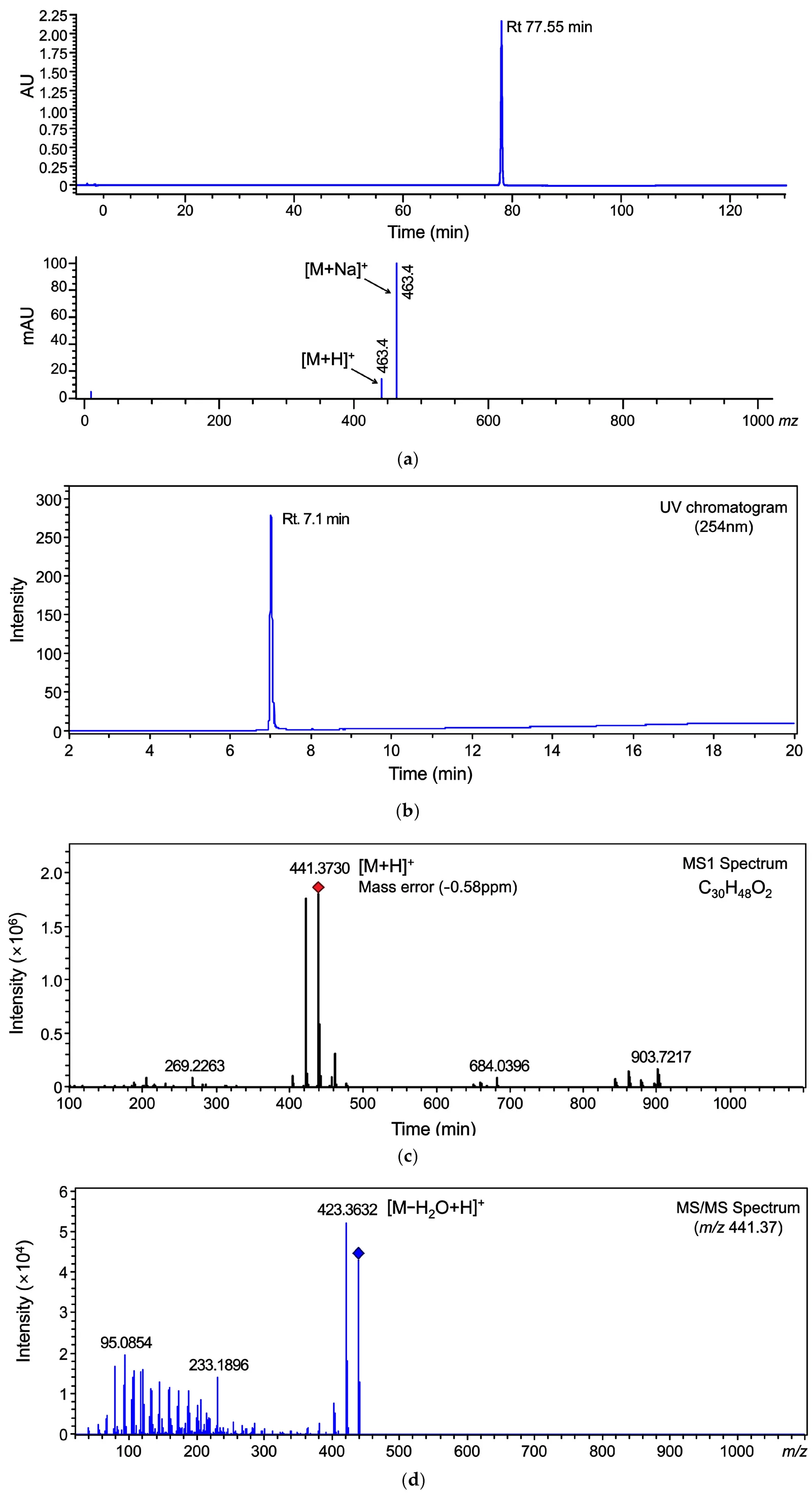

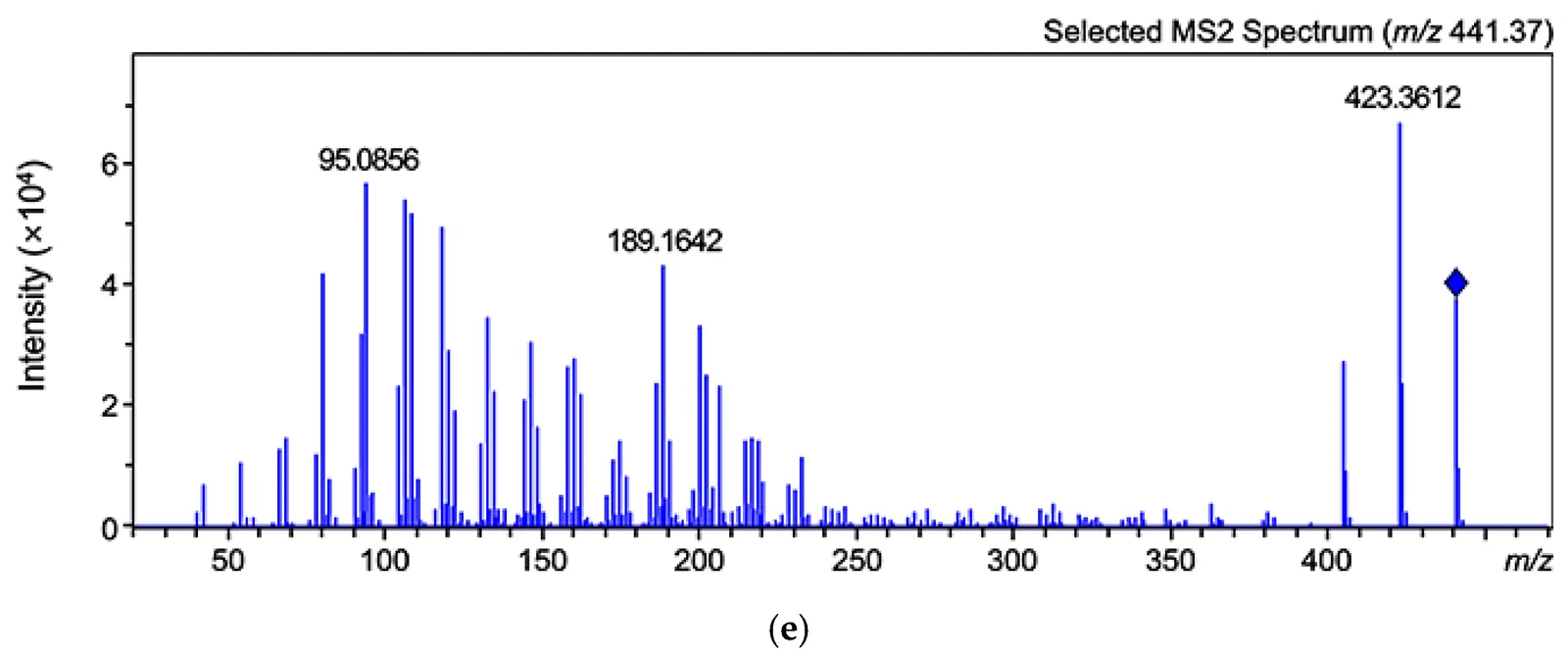
LC–ESI–MS and HR-MS/MS analysis of the isolated compound from *G. lucidum* mycelia. (**a**) LC–ESI–MS chromatogram of the isolated compound showing a dominant peak at a retention time of 77.55 min and the corresponding mass spectrum with ions at *m*/*z* 441.4 [M + H]^+^ and 463.4 [M + Na]^+^. (**b**) UV chromatogram monitored at 254 nm showing a dominant peak at 7.1 min. (**c**) HR-MS spectrum showing the protonated molecular ion at *m*/*z* 441.3730 [M + H]^+^, corresponding to the molecular formula C_30_H_48_O_2_. (**d**) MS/MS spectrum of the precursor ion at *m*/*z* 441.37 showing a major fragment ion at *m*/*z* 423.3632 [M − H_2_O + H]^+^. (**e**) Selected MS/MS spectrum showing fragment ions at *m*/*z* 423.3612, 189.1642, and 95.0856.

LC–ESI–MS and HR-MS/MS analyses were performed to determine the molecular weight and molecular formula of the compound isolated from the final purified fraction. In the LC–ESI–MS analysis, the isolated compound showed a dominant chromatographic peak at a retention time of 77.55 min. The mass spectrum corresponding to this peak displayed two characteristic ions at *m*/*z* 441.4 and 463.4, which were assigned to the protonated molecular ion [M + H]^+^ and sodium adduct ion [M + Na]^+^, respectively. These data indicated that the isolated compound had an approximate molecular weight of 440 Da (Figure 3a).

High-resolution mass spectrometry was then conducted to further confirm the exact mass and molecular formula of the isolated compound. The UV chromatogram monitored at 254 nm showed a single dominant peak at a retention time of 7.1 min, supporting the presence of a major purified compound. In the HR-MS spectrum, the protonated molecular ion [M + H]^+^ was observed at *m*/*z* 441.3730. This value was consistent with the calculated mass of C_30_H_49_O_2_^+^ and supported the molecular formula C_30_H_48_O_2_, with a mass error of −0.58 ppm (Figure 3b,c).

To obtain additional structural information, MS/MS analysis was performed using the precursor ion at *m*/*z* 441.37. The MS/MS spectrum showed a major fragment ion at *m*/*z* 423.3632, corresponding to [M − H_2_O + H]^+^, indicating the loss of water from the protonated molecule. This dehydration fragment suggested the presence of hydroxyl functionality in the isolated compound. Additional fragment ions at *m*/*z* 189.1642 and 95.0856 were also detected, which may be associated with partial fragmentation of the triterpenoid skeleton. Together, the LC–ESI–MS and HR-MS/MS data support that the isolated compound is a hydroxylated triterpenoid-like molecule with the molecular formula C_30_H_48_O_2_ (Figure 3d,e).

### 3.3. Spectroscopic Characterization and Identification of the Isolated Compound as Polycarpol

The isolated compound was initially obtained as an unknown major compound from Fr.4.4.5 and subjected to spectroscopic characterization. LC–ESI–MS, HR-MS/MS, and NMR analyses were performed to determine its chemical structure. The spectroscopic data revealed that the isolated compound was a hydroxylated triterpenoid with the molecular formula C_30_H_48_O_2_. After the structural analysis was completed, comparison with previously reported data confirmed that the compound corresponded to polycarpol, a known triterpenoid compound.

The ^1^H NMR spectrum showed characteristic olefinic proton signals at δ_H_ 6.19, 5.96–6.06, and 5.56–5.75, assigned to H-4, H-16, and H-25, respectively (Figure S1). The ^13^C NMR spectrum exhibited 30 carbon resonances, supporting a triterpenoid-type carbon framework (Figure S2). The DEPT-135 and HSQC spectra allowed classification of the carbon signals into methyl, methylene, methine, and quaternary carbons (Figures S3 and S4). The olefinic proton signals were correlated with olefinic carbons at δ_C_ 129.3, 130.0, and 129.4 in the HSQC spectrum, indicating the presence of multiple double bonds in the structure (Table 1 and Table S2).

In addition to the olefinic signals, oxygenated methine protons were observed at δ_H_ 4.09–4.20 and 4.16–4.20, corresponding to H-10 and H-30, respectively. These protons showed direct HSQC correlations with oxygenated carbons at δ_C_ 72.9 and 70.5, supporting the presence of hydroxylated methine carbons (Table S2). Another deshielded carbon signal at δ_C_ 70.4 was assigned to C-6, which was associated with H-6 at δ_H_ 3.67. The aliphatic region contained multiple methyl and methylene proton signals between δ_H_ 0.85 and 2.26, consistent with a highly substituted tetracyclic triterpenoid skeleton.

The COSY spectrum provided key proton–proton correlations that defined several partial spin systems (Figure S5). The correlations of H-4/H_2_-5/H-6 supported the connectivity around the C3=C4 double bond and adjacent ring methylene/methine region. Additional COSY correlations, including H-10/H_2_-11/H_2_-12, H-16/H_2_-17, H-20/H-21, H-21/H_2_-23/H_2_-24/H-25, and H_2_-29/H-30, supported the arrangement of the tetracyclic core and side-chain moieties (Table S2).

**Figure 4:**
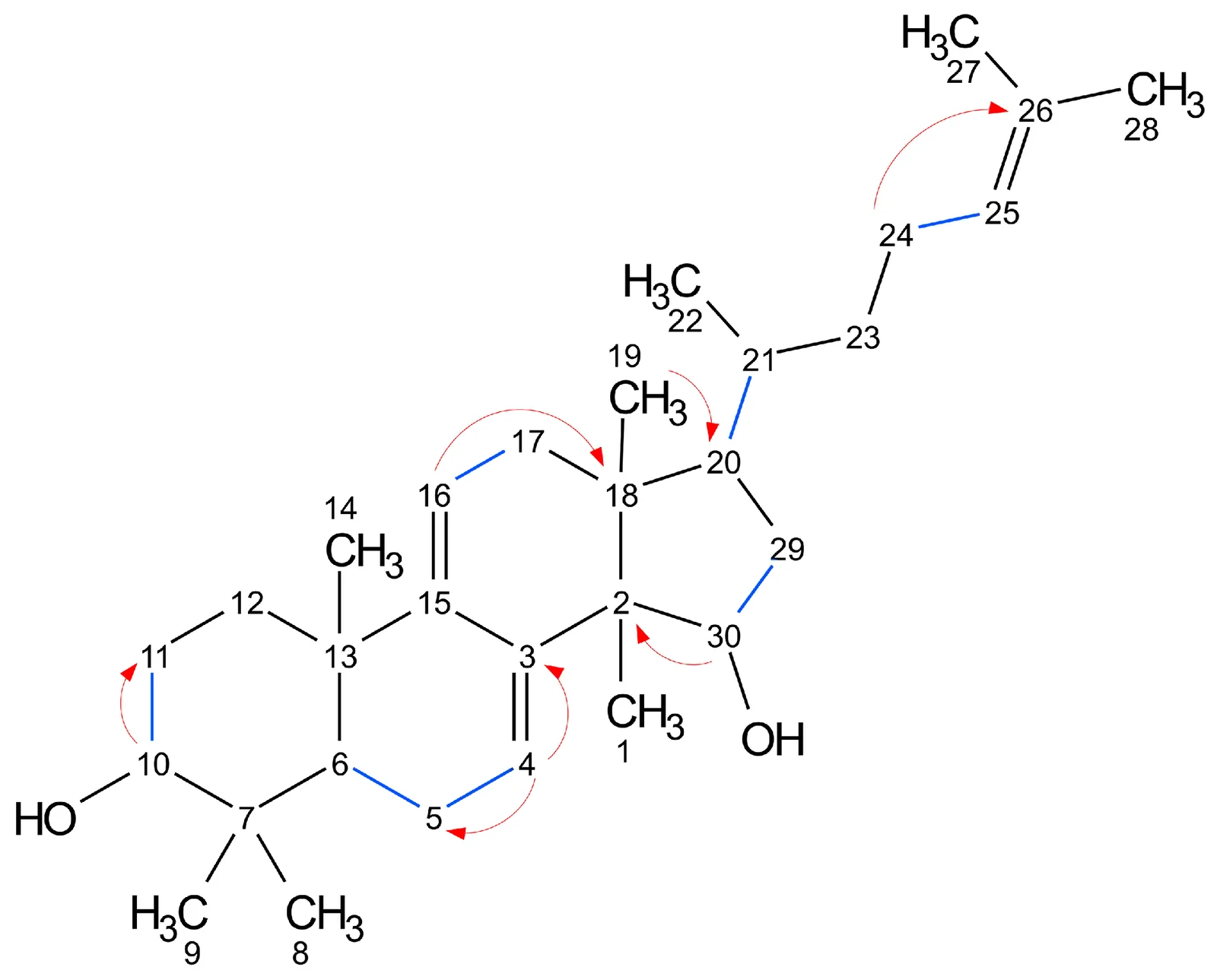
Key two-dimensional NMR correlations of the isolated compound. Key COSY and HMBC correlations used for structural elucidation of the isolated compound. Blue lines indicate selected COSY correlations, and red arrows indicate selected HMBC correlations.

Long-range HMBC correlations further established the carbon skeleton and the positions of key functional groups (Figure S6). The HMBC correlation from H-30 to C-2 indicated the linkage of the oxygenated methine C-30 to the ring system. Correlations from H-4 to C-5 and from H-6 to C-4 supported the C3=C4 olefinic region, while correlations from H-16 to C-17 and C-18 supported the C15=C16 double bond and adjacent ring connectivity. In the side chain, HMBC correlations from H_2_-24 to C-25 and C-26 and from H-25 to C-24, C-27, and C-28 supported the presence of the C25=C26 double bond bearing methyl groups at C-27 and C-28. Furthermore, correlations involving H_3_-19, H_3_-22, and H_2_-29 supported the connectivity around C-20 and the ring D/side-chain junction (Table 2 and Table S2, Figure 4).

Collectively, the LC–ESI–MS, HR-MS/MS, and NMR data established the structure of the isolated compound as a hydroxylated triterpenoid with the molecular formula C_30_H_48_O_2_. After completion of the spectroscopic analysis, the obtained structural and spectroscopic features were found to be consistent with those previously reported for polycarpol. Therefore, the isolated compound was finally identified as polycarpol, a known triterpenoid compound obtained from *G. lucidum* mycelia.

### 3.4. Effect of Polycarpol on B16F10 Cell Viability

The effect of polycarpol on B16F10 cell viability was evaluated to determine suitable treatment concentrations for subsequent melanogenesis-related assays. B16F10 cells were treated with polycarpol at the indicated concentrations, and cell viability was measured using the MTT assay.

**Figure 5:**
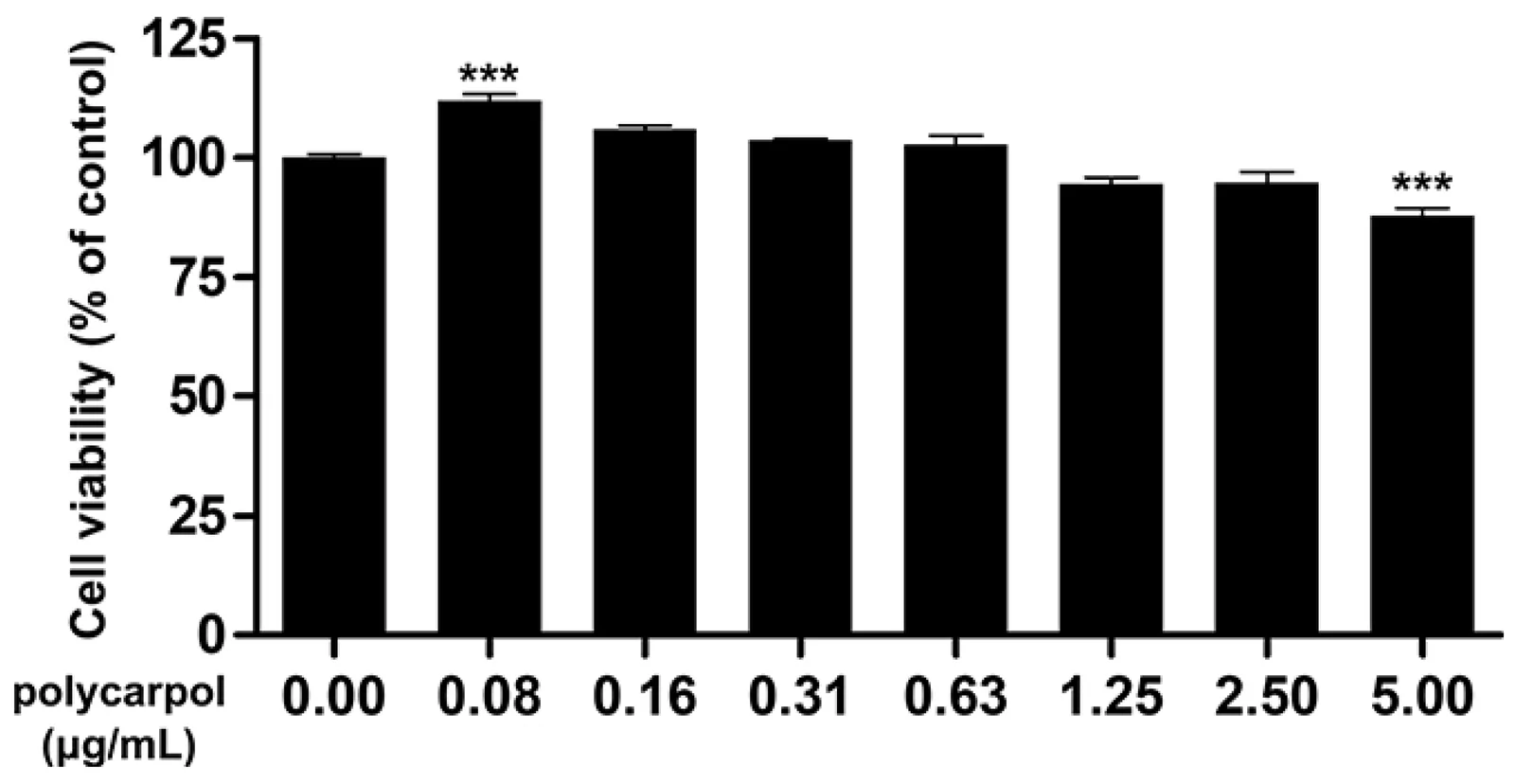
Effect of polycarpol on the viability of B16F10 melanoma cells. B16F10 cells were treated with polycarpol at the indicated concentrations, and cell viability was assessed using the MTT assay. Cell viability was expressed as a percentage of the untreated control. Data are presented as the mean ± SD. Statistical significance was determined by one-way ANOVA followed by Tukey’s test. *** *p* < 0.001 compared with untreated B16F10 cells.

Polycarpol did not significantly reduce cell viability at concentrations up to 2.5 μg/mL, whereas a significant reduction was observed at 5 μg/mL (11.35 μM). Based on these results, 1 μg/mL (2.27 μM) and 2 μg/mL (4.54 μM) were selected as the treatment concentrations for subsequent experiments, including cellular tyrosinase activity, melanin content, and Western blot analyses (Figure 5).

### 3.5. Effects of Polycarpol on α-MSH-Induced Tyrosinase Activity and Melanin Production

**Figure 6:**
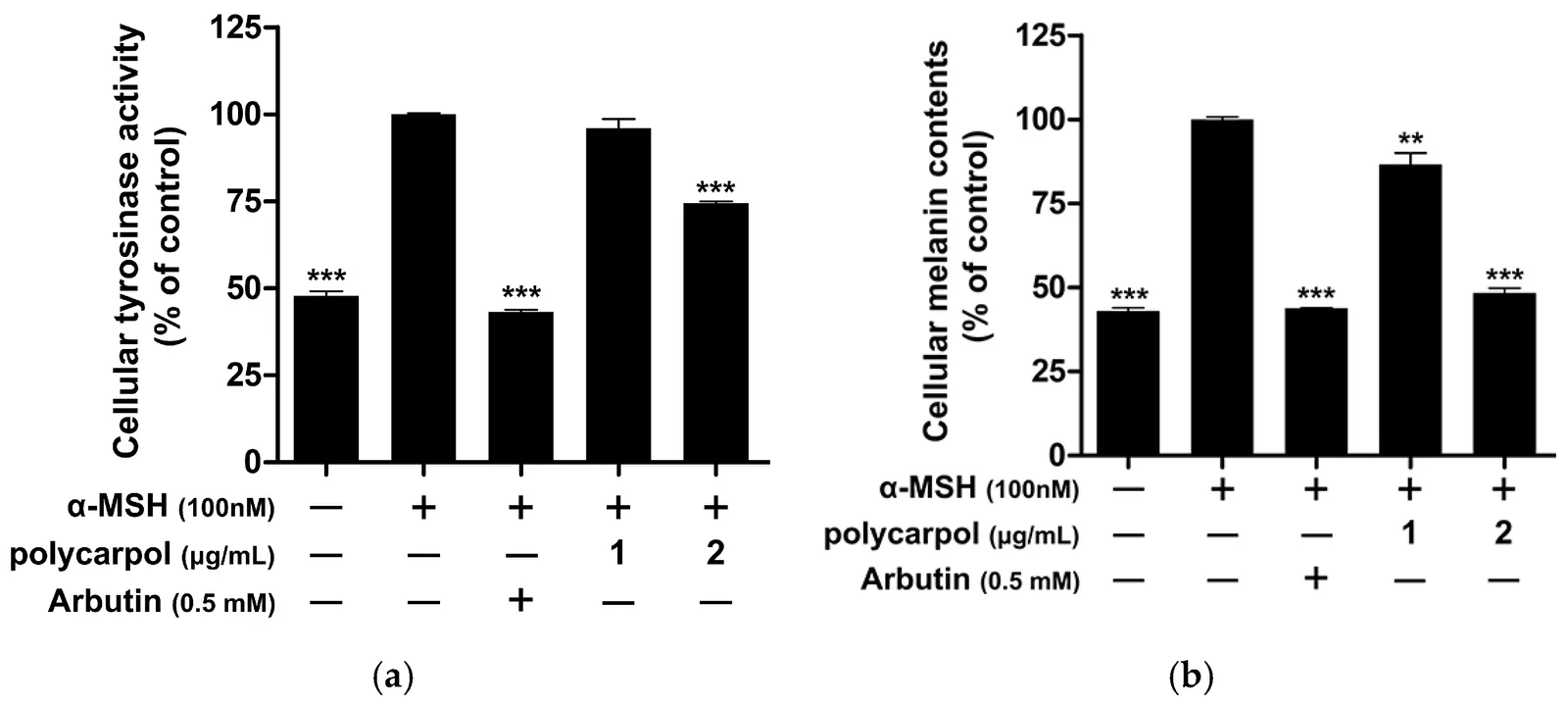
Effects of polycarpol on α-MSH-induced cellular tyrosinase activity (**a**) and melanin content (**b**) in B16F10 melanoma cells. B16F10 cells were stimulated with 100 nM α-MSH and treated with polycarpol at 1 μg/mL (2.27 μM) and 2 μg/mL (4.54 μM). Arbutin at 0.5 mM was used as a positive control. Data are presented as the mean ± SD. Statistical significance was determined by one-way ANOVA followed by Tukey’s test. ** *p* < 0.01 and *** *p* < 0.001 compared with the α-MSH-treated control group.

The anti-melanogenic activity of polycarpol was evaluated by measuring cellular tyrosinase activity and melanin content in α-MSH-stimulated B16F10 melanoma cells. Treatment with α-MSH markedly increased cellular tyrosinase activity compared with untreated control cells, confirming the induction of melanogenic activity. However, polycarpol treatment significantly reduced α-MSH-induced tyrosinase activity at both 1 and 2 μg/mL (Figure 6a). This result indicates that polycarpol can suppress a key enzymatic step involved in melanin biosynthesis.

Melanin content was then measured to determine whether the reduction in tyrosinase activity was accompanied by decreased melanin accumulation. Consistent with the tyrosinase activity results, α-MSH treatment increased cellular melanin content, whereas polycarpol significantly reduced α-MSH-induced melanin production. In particular, the melanin content in cells treated with 2 μg/mL polycarpol was reduced to a level similar to that observed in the arbutin-treated group. This suggests that polycarpol effectively attenuates α-MSH-induced melanin synthesis at a low concentration.

Because 1 and 2 μg/mL polycarpol did not markedly affect B16F10 cell viability, the observed decrease in tyrosinase activity and melanin content is likely attributable to the anti-melanogenic activity of polycarpol rather than a nonspecific reduction in cell number. Together, these findings demonstrate that polycarpol suppresses α-MSH-induced melanogenesis in B16F10 cells, with the 2 μg/mL treatment showing a melanin-lowering effect similar to arbutin (Figure 6b).

### 3.6. Effects of Polycarpol on Melanogenesis-Related Protein Expression

**Figure 7:**
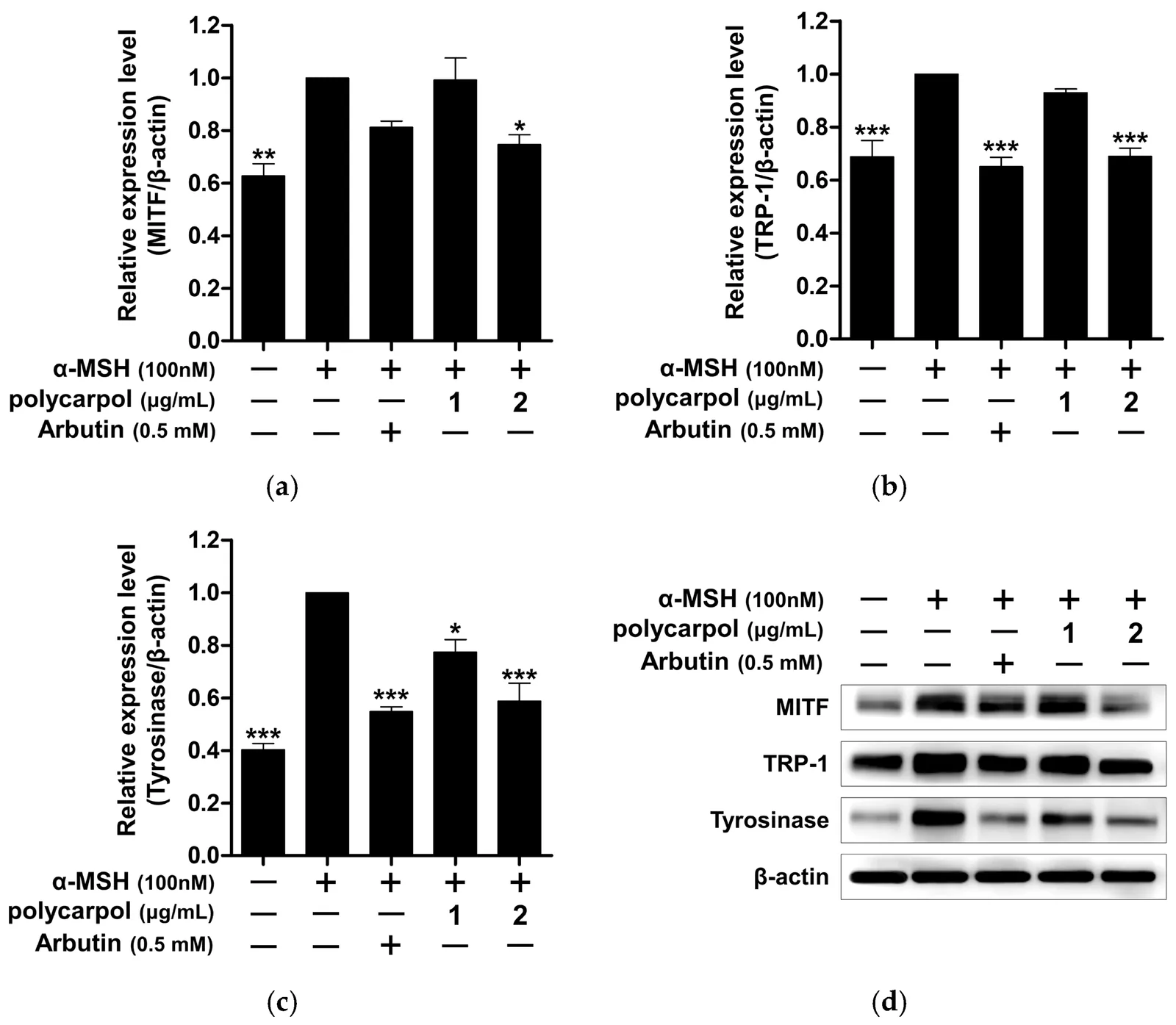
Effects of polycarpol on melanogenesis-related protein expression in α-MSH-stimulated B16F10 cells. B16F10 cells were stimulated with 100 nM α-MSH and treated with polycarpol at 1 or 2 μg/mL. Arbutin at 0.5 mM was used as a positive control. Protein expression levels of MITF (**a**), TRP-1 (**b**), and tyrosinase (**c**) were determined by Western blot analysis (**d**). β-Actin was used as the loading control. Quantitative data are presented as the mean ± SD. Statistical significance was determined by one-way ANOVA followed by Tukey’s test. * *p* < 0.05, ** *p* < 0.01, and *** *p* < 0.001 compared with the α-MSH-treated control group.

To further investigate the mechanism underlying the anti-melanogenic effect of polycarpol, the expression levels of melanogenesis-related proteins were examined by Western blot analysis. In α-MSH-stimulated B16F10 cells, the expression of microphthalmia-associated transcription factor (MITF), tyrosinase, and tyrosinase-related protein-1 (TRP-1) was increased compared with untreated control cells (Figure 7). This result confirmed that α-MSH stimulation activated the melanogenic protein expression program in B16F10 cells.

Treatment with polycarpol reduced the α-MSH-induced expression of MITF, tyrosinase, and TRP-1. In particular, polycarpol at 2 μg/mL clearly decreased the expression levels of these melanogenic proteins. The reduction in MITF expression suggests that polycarpol may suppress melanogenesis by regulating an upstream transcriptional regulator of melanogenic enzyme expression. Consistently, the decreased levels of tyrosinase and TRP-1 support the inhibitory effects of polycarpol on cellular tyrosinase activity and melanin production observed in the previous assays.

Arbutin, used as a positive control, also reduced the expression of melanogenesis-related proteins. The polycarpol-treated groups showed a suppressive pattern similar to the arbutin-treated group, particularly at 2 μg/mL. These results indicate that polycarpol attenuates α-MSH-induced melanogenesis at least in part by downregulating MITF and its downstream melanogenic proteins, including tyrosinase and TRP-1 (Figure 7).

### 3.7. Effects of Polycarpol on CREB and MAPK Signaling Pathways

**Figure 8:**
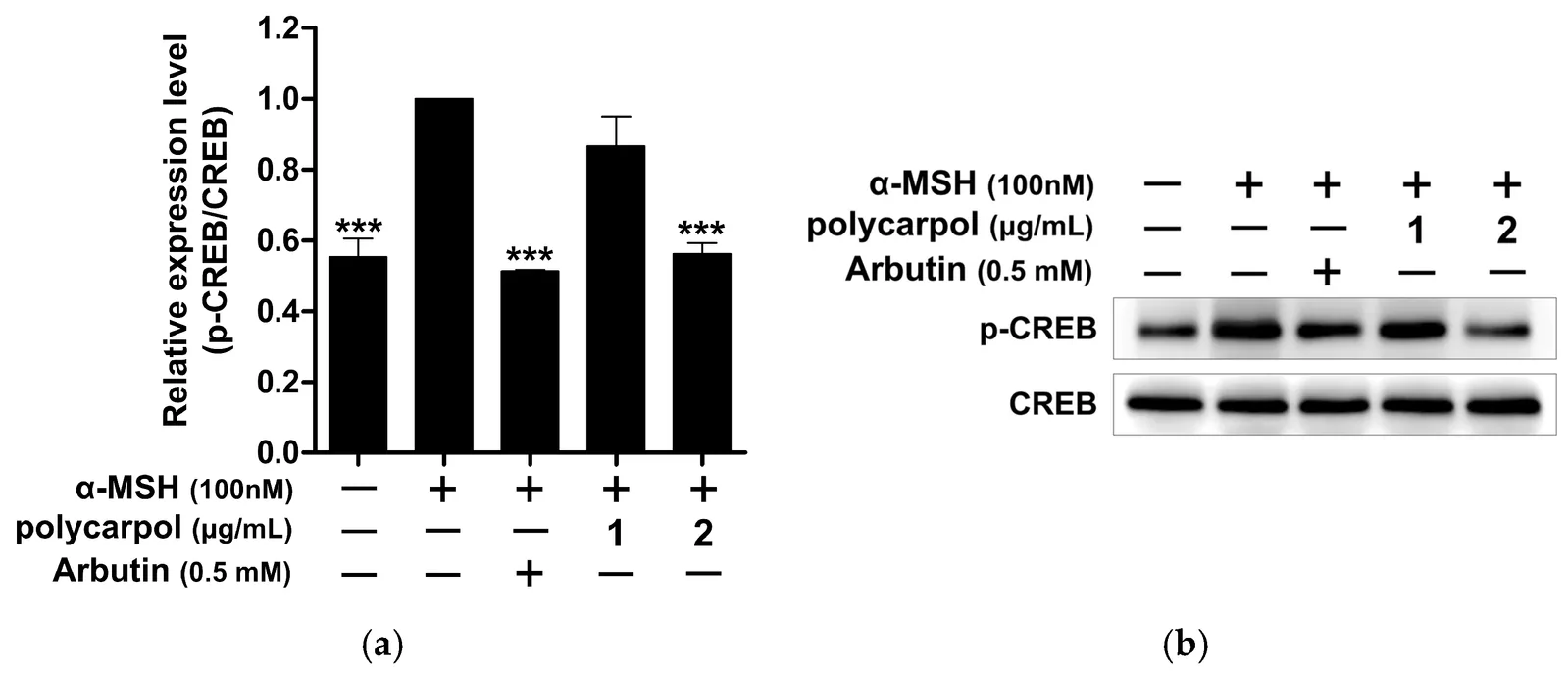
Effect of polycarpol on CREB phosphorylation in α-MSH-stimulated B16F10 cells. B16F10 cells were stimulated with 100 nM α-MSH and treated with polycarpol at 1 or 2 μg/mL. Arbutin at 0.5 mM was used as a positive control. CREB and phosphorylated CREB expression levels (**a**) were analyzed by Western blotting (**b**). The relative p-CREB/CREB ratio was quantified. Data are presented as the mean ± SD. Statistical significance was determined by one-way ANOVA followed by Tukey’s test. *** *p* < 0.001 compared with the α-MSH-treated control group.

To further investigate the upstream signaling events associated with the anti-melanogenic effect of polycarpol, the phosphorylation levels of CREB and MAPK signaling molecules were examined by Western blot analysis. In α-MSH-stimulated B16F10 cells, CREB phosphorylation was increased compared with untreated control cells, indicating activation of CREB-mediated melanogenic signaling. Treatment with polycarpol reduced the α-MSH-induced increase in p-CREB levels. In particular, polycarpol at 2 μg/mL markedly decreased the p-CREB/CREB ratio, suggesting that polycarpol may suppress melanogenesis by attenuating CREB activation (Figure 8).

**Figure 9:**
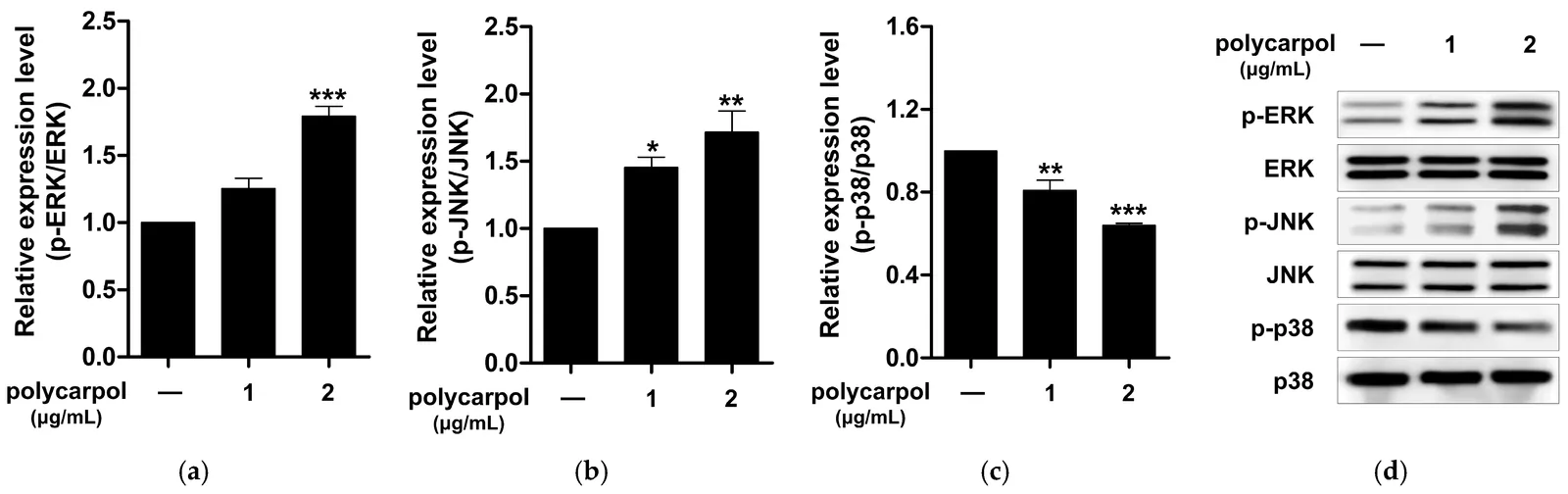
Effect of polycarpol on MAPK signaling molecules in B16F10 cells. B16F10 cells were treated with polycarpol at 1 or 2 μg/mL, and the phosphorylation levels of ERK, JNK, and p38 were determined by Western blot analysis. (**a**) Relative p-ERK/ERK expression level. (**b**) Relative p-JNK/JNK expression level. (**c**) Relative p-p38/p38 expression level. (**d**) Representative Western blot bands for p-ERK, ERK, p-JNK, JNK, p-p38, and p38. Data are presented as the mean ± SD. Statistical significance was determined by one-way ANOVA followed by Tukey’s test. * *p* < 0.05, ** *p* < 0.01, and *** *p* < 0.001 compared with untreated B16F10 cells.

The effects of polycarpol on MAPK signaling were also examined by analyzing the phosphorylation levels of ERK, JNK, and p38. Polycarpol treatment increased ERK and JNK phosphorylation but decreased p38 phosphorylation compared with untreated cells. The increase in phosphorylated MAPK levels was more evident at 2 μg/mL polycarpol. Because MAPK signaling can regulate MITF expression and stability, these results suggest that polycarpol-induced modulation of MAPK pathways may be associated with the reduced expression of MITF and its downstream melanogenic proteins observed in B16F10 cells (Figure 9).

Taken together, these results indicate that polycarpol suppresses α-MSH-induced CREB phosphorylation and modulates MAPK signaling pathways. These signaling changes may contribute to the downregulation of MITF, tyrosinase, and TRP-1, ultimately leading to reduced cellular tyrosinase activity and melanin production.

## 4. Discussion

In the present study, polycarpol was isolated from cultured *G. lucidum* mycelia, and its anti-melanogenic activity was evaluated in B16F10 murine melanoma cells. Previous studies have primarily focused on fruiting bodies and spores, which are traditionally used materials and well-known sources of bioactive compounds [2], whereas the mycelial stage remains relatively underexplored as a source of anti-melanogenic secondary metabolites. Because mycelia can be produced under controlled submerged culture conditions, they provide a reproducible fungal material for investigating secondary metabolites [15]. Together with previous reports of triterpenoids in *G. lucidum* mycelia, including ganoderic acid derivatives [16], the isolation of polycarpol supports the capacity of cultured *G. lucidum* mycelia to produce biologically active triterpenoids.

Spectroscopic analyses and comparison with previously reported data identified the isolated compound as polycarpol, a triterpenoid previously reported from several plant species of the Annonaceae family [17]. Although its chemotaxonomic significance and antimicrobial activity have been described, reports on its anti-melanogenic activity remain limited. The present findings therefore provide evidence that polycarpol suppresses melanogenesis in B16F10 cells.

Melanogenesis is primarily regulated by MITF and its downstream proteins, including tyrosinase and tyrosinase-related proteins [18,19]. In the present study, polycarpol reduced α-MSH-induced cellular tyrosinase activity and melanin content at 1 and 2 μg/mL, concentrations that did not markedly affect cell viability. At 2 μg/mL, polycarpol reduced melanin content to a level similar to that observed with 0.5 mM arbutin under the tested conditions [20]. Polycarpol also decreased the expression of MITF, tyrosinase, and TRP-1, consistent with previous studies showing that suppression of MITF reduces the expression of downstream melanogenic proteins [10]. These findings suggest that the anti-melanogenic activity of polycarpol is associated with reduced MITF-related melanogenic protein expression and is unlikely to be explained solely by nonspecific cytotoxicity.

Other *G. lucidum*-derived components, including polysaccharides, have also been reported to suppress melanogenesis through cAMP/PKA, ROS/MAPK, and paracrine signaling pathways [21,22]. More directly relevant to the present study, the *G. lucidum*-derived triterpenoids ganodermanondiol and ganodermanontriol have shown anti-melanogenic activity in B16F10 cells [23,24]. Ganodermanontriol reduced melanin content and the expression of MITF, tyrosinase, and TRP-1 while altering CREB and MAPK phosphorylation [24]. The present study extends these findings by demonstrating that polycarpol, obtained from cultured *G. lucidum* mycelia, also exhibits anti-melanogenic activity. Further studies are required to determine the structure–activity relationships among these triterpenoids.

CREB and MAPK signaling pathways are involved in the regulation of melanogenesis. Activation of the cAMP/PKA/CREB axis promotes MITF transcription and the expression of melanogenic enzymes [25], whereas suppression of CREB phosphorylation has been associated with reduced MITF expression [10,22]. In the present study, polycarpol decreased α-MSH-induced CREB phosphorylation, suggesting that reduced CREB activation may contribute to the decreased expression of MITF and its downstream proteins. Polycarpol increased ERK and JNK phosphorylation but decreased p38 phosphorylation. Previous work showed that ganodermanontriol increased ERK and JNK phosphorylation but reduced p38 phosphorylation [24]. Because MAPK effects on melanogenesis vary according to the kinase branch and cellular context, the observed changes should be interpreted as being associated with, rather than directly responsible for, the anti-melanogenic activity of polycarpol. Studies using pathway-specific inhibitors or gene-silencing approaches are required to establish causal relationships.

This study has several limitations. First, the findings should be validated in human melanocytes or reconstructed skin models. Second, the direct molecular targets of polycarpol and the mechanisms underlying the observed changes in CREB and MAPK phosphorylation remain unclear. Third, the low isolated yield of polycarpol limits conclusions regarding its practical production, and quantitative profiling and optimization of culture and recovery conditions are required. Overall, the present study demonstrates that cultured *G. lucidum* mycelia can produce polycarpol, an anti-melanogenic triterpenoid.

## 5. Conclusions

This study demonstrates that cultured *G. lucidum* mycelia can produce polycarpol, a triterpenoid with anti-melanogenic activity. In α-MSH-stimulated B16F10 cells, polycarpol reduced cellular tyrosinase activity, melanin content, and the expression of MITF, tyrosinase, and TRP-1 at concentrations that did not significantly affect cell viability. The observed changes in CREB and MAPK phosphorylation suggest that its anti-melanogenic activity is associated with MITF-related melanogenic signaling. However, the low isolated yield and the use of a single murine cell model warrant further optimization of production conditions and validation in additional pigmentation models.

## Figures and Tables

**Table 1:** ^1^H and ^13^C NMR spectroscopic data of polycarpol.

Position	δ_C_ (ppm)	δ_H_ (ppm)	Carbon Type	DEPT-135
C1	22.4	0.87–1.05	CH_3_	+
C2	45.3	—	C	—
C3	130.9	—	C	—
C4	129.3	6.19	CH	+
C5	45	1.48–1.61	CH_2_	−
C6	70.4	3.67	CH	+
C7	38.5	—	C	—
C8	22.4	0.85	CH_3_	+
C9	22.4	0.9	CH_3_	+
C10	72.9	4.09–4.20	CH	+
C11	33.5	1.48–1.61	CH_2_	−
C12	31.8	1.48–1.61	CH_2_	−
C13	50.2	—	C	—
C14	23.1	0.95	CH_3_	+
C15	135.7	—	C	—
C16	130	5.96–6.06	CH	+
C17	32.6	1.83–1.90	CH_2_	−
C18	50.5	—	C	—
C19	20.1	1.05	CH_3_	+
C20	40.2	1.62–1.69	CH	+
C21	35.5	1.62–1.69	CH	+
C22	20.3	1.62–1.69	CH_3_	+
C23	29.8	1.48–1.61	CH_2_	−
C24	28.7	1.48–1.61	CH_2_	−
C25	129.4	5.56–5.75	CH	+
C26	135.8	—	C	—
C27	18.2	1.60–1.65	CH_3_	+
C28	17.5	1.60–1.65	CH_3_	+
C29	29	2.17–2.26	CH_2_	−
C30	70.5	4.16–4.20	CH	+

**Table 2:** 2D NMR correlations and structural assignments of polycarpol.

Position	HSQC Assignment	Key COSY Correlations	Key HMBC Correlations	Structural Assignment
C1	H_3_-1/C-1	—	—	Angular methyl attached to C2
C2	—	—	H-30 → C-2	Ring junction carbon
C3	—	—	—	Olefinic ring-junction carbon
C4	H-4/C-4	H-4 ↔ H_2_-5	H-4 → C-5	Proton-bearing carbon of the C3=C4 double bond
C5	H_2_-5/C-5	H_2_-5 ↔ H-4, H-6	H-4 → C-5	Ring methylene adjacent to the C3=C4 double bond
C6	H-6/C-6	H-6 ↔ H_2_-5	H-6 → C-4	Deshielded ring-junction methine carbon
C7	—	—	—	Quaternary carbon bearing Me-8 and Me-9
C8	H_3_-8/C-8	—	—	Gem-dimethyl methyl at C7
C9	H_3_-9/C-9	—	—	Gem-dimethyl methyl at C7
C10	H-10/C-10	H-10 ↔ H_2_-11	H-10 → C-11, C-12	Oxygenated methine carbon
C11	H_2_-11/C-11	H_2_-11 ↔ H-10, H_2_-12	H-10 → C-11	Ring methylene adjacent to C10
C12	H_2_-12/C-12	H_2_-12 ↔ H_2_-11	H-10 → C-12	Ring methylene in ring A
C13	—	—	—	Ring junction carbon
C14	H_3_-14/C-14	—	—	Angular methyl attached to C13
C15	—	—	—	Olefinic quaternary carbon of the C15=C16 double bond
C16	H-16/C-16	H-16 ↔ H_2_-17	H-16 → C-17, C-18	Proton-bearing carbon of the C15=C16 double bond
C17	H_2_-17/C-17	H_2_-17 ↔ H-16	H-16 → C-17	Allylic methylene adjacent to C15=C16
C18	—	—	H-16 → C-18; H_3_-19 → C-18	Ring junction carbon
C19	H_3_-19/C-19	—	H_3_-19 → C-18, C-20	Angular methyl attached to C18
C20	H-20/C-20	H-20 ↔ H-21, H_2_-29	H_3_-19 → C-20; H_3_-22 → C-20; H_2_-29 → C-20	Ring D/side-chain junction methine
C21	H-21/C-21	H-21 ↔ H-20, H_2_-23	H_3_-22 → C-21	Side-chain methine bearing Me-22
C22	H_3_-22/C-22	—	H_3_-22 → C-20, C-21	Methyl attached to C21
C23	H_2_-23/C-23	H_2_-23 ↔ H-21, H_2_-24	—	Side-chain methylene
C24	H_2_-24/C-24	H_2_-24 ↔ H_2_-23, H-25	H_2_-24 → C-25, C-26	Allylic methylene adjacent to C25=C26
C25	H-25/C-25	H-25 ↔ H_2_-24	H-25 → C-24, C-27, C-28	Proton-bearing carbon of the C25=C26 double bond
C26	—	—	H_2_-24 → C-26	Olefinic quaternary carbon of the C25=C26 double bond
C27	H_3_-27/C-27	—	H-25 → C-27	Olefinic methyl attached to C26
C28	H_3_-28/C-28	—	H-25 → C-28	Olefinic methyl attached to C26
C29	H_2_-29/C-29	H_2_-29 ↔ H-20, H-30	H_2_-29 → C-20, C-30	Methylene adjacent to C30
C30	H-30/C-30	H-30 ↔ H_2_-29	H_2_-29 → C-30; H-30 → C-2	Oxygenated methine carbon

## Data Availability

The data supporting the findings of this study are available within the article and its Supplementary Materials. Additional data are available from the corresponding author upon reasonable request.

## Supplementary Materials

The following supporting information can be downloaded at: https://www.mdpi.com/article/10.3390/jof12080625/s1, Figure S1: ^1^H NMR spectrum of polycarpol. The spectrum was recorded in CDCl_3_ using an 800 MHz NMR spectrometer. Characteristic olefinic and oxygenated methine proton signals are indicated; Figure S2: ^13^C NMR spectrum of polycarpol. The ^13^C NMR spectrum shows 30 carbon resonances, including olefinic, oxygenated, and aliphatic carbon signals; Figure S3: DEPT-135 spectrum of polycarpol. The DEPT-135 spectrum was used to distinguish CH/CH_3_, CH_2_, and quaternary carbon signals; Figure S4: HSQC spectrum of polycarpol. The HSQC spectrum shows direct ^1^H–^13^C correlations used for protonated carbon assignment; Figure S5: COSY spectrum of polycarpol. The COSY spectrum shows selected proton–proton correlations supporting partial spin systems of polycarpol; Figure S6: HMBC spectrum of polycarpol. The HMBC spectrum shows long-range ^1^H–^13^C correlations supporting the structural assignment of polycarpol; Table S1: HPLC gradient system used for chromatographic analysis; Table S2: Complete ^1^H, ^13^C, DEPT-135, HSQC, COSY, and HMBC NMR assignments of polycarpol isolated from *G. lucidum* mycelia.
